# Cytokines and Metabolic Patterns in Pediatric Patients with Critical Illness

**DOI:** 10.1155/2010/354047

**Published:** 2010-05-16

**Authors:** George Briassoulis, Shekhar Venkataraman, Ann Thompson

**Affiliations:** ^1^Division of Pediatric Critical Care Medicine, Children's Hospital of Pittsburgh of UPMC, 3705 Fifth Avenue, Pittsburgh, PA 15213, USA; ^2^Pediatric Intensive Care Unit, University Hospital of Heraklion, University of Crete, Voutes Area, Heraklion, 71110 Crete, Greece

## Abstract

It is not known if cytokines, which are cell-derived mediators released during the host immune response to stress, affect metabolic response to stress during critical illness. The aim of this prospective study was to determine whether the metabolic response to stress is related to the inflammatory interleukin-6 (IL-6), 10 (IL-10), and other stress mediators' responses and to assess their relationships with different feeding patterns, nutritional markers, the severity of illness as assessed by the Multiple Organ System Failure (MOSF), the Pediatric Risk of Mortality Score (PRISM), systemic inflammatory response syndrome (SIRS), and mortality in critically ill children. Patients were classified as hypermetabolic, normometabolic, and hypometabolic when the measured resting energy expenditures (REE) were >110%, 90–110% and, <90% of the predicted basal metabolic rate, respectively. The initial predominance of the hypometabolic pattern (48.6%) declined within 1 week of acute stress (20%), and the hypermetabolic patterns dominated only after 2 weeks (60%). Only oxygen consumption (VO_2_) and carbon dioxide production (VCO_2_) (*P* < .0001) but none of the cytokines and nutritional markers, were independently associated with a hypometabolic pattern. REE correlated with the IL-10 but not PRISM. In the presence of SIRS or sepsis, CRP, IL-6, IL-10, Prognostic Inflammatory and Nutritional Index (NI), and triglycerides—but not glucose, VO_2_, or VCO_2_ increased significantly. High IL-10 levels (*P* = .0000) and low measured REE (*P* = .0000) were independently associated with mortality (11.7%), which was higher in the hypometabolic compared to other metabolic patterns (*P* < .005). Our results showed that only VO_2_ and VCO_2_, but not IL-6 or IL-10, were associated with a hypometabolic pattern which predominated the acute phase of stress, and was associated with increased mortality. Although in SIRS or sepsis, the cytokine response was reliably reflected by increases in NI and triglycerides, it was different from the metabolic (VO_2_, VCO_2_) or glucose response.

## 1. Introduction

The relationship between cytokines, acute-phase reactants, organ failure, and energy expenditure in critically ill children are poorly understood [1–3]. Interleukin-6 (IL-6) has been reported to act synergistically with glucocorticoids in stimulating the production of acute-phase reactants [4]. However, cytokines consist only a part of the deranged metabolism during stress in children. A number of acute-phase proteins, such as C-reactive protein (CRP) and fibrinogen, rise after serious injury or sepsis in association with a drop in serum albumin, transthyretin, and transferrin. The aims of this prospective study were to determine the relationship between inflammatory cytokines and other stress mediators on the following: (1) measured energy expenditure, (2) metabolic pattern, (3) nutritional markers, (4) feeding patterns, (5) severity of illness, and (6) mortality in critically ill children.

## 2. Materials and Methods

### 2.1. Patients

A first part of this prospective study was performed in the tertiary care Pediatric Intensive Care Unit (PICU) at Pittsburgh's Children Hospital in 1997 when data were measured and recorded longitudinally (37 patients), and a second part in the tertiary care PICU at the University Hospital of Crete between 2006–2008, when only one measurement was performed per patient (40 patients). First day results did not differ between the two parts and the data were pooled for analysis. Repeated measurements were restricted to the first part data. This study was approved by the Institutional Review Boards, and informed consent was obtained from parents. All patients who were admitted to the PICU and mechanically ventilated (MV) for at least 24 hours, were eligible for the study. Patients who were intubated with cuffed endotracheal tubes and those with uncuffed endotracheal tubes without a significant air-leak (difference between inspired and expired tidal volume <10%) were included. All patients were ventilated using the Siemens-Elema Servo 900C ventilator in both PICUs, and all measurements were done by the same investigator (GB) using the Deltatrac metabolic monitor. Children with chromosomal anomalies, dysmorphic syndromes, and severe brain injury, being evaluated for brain death, were excluded from the study. Children who required fraction of inspired oxygen (FIO_2_) >0.6, use of continuous or bias flow, inspiratory gas containing gases other than air and oxygen, or who had chest tubes or a bronchopleural fistula were also excluded. All patients were hemodynamically stable before the study period. The physician clinically responsible for the patient made decisions regarding caloric intake and content entirely independent of the study.

### 2.2. Data Collected

Data collected included demographics, clinical diagnoses, and vital signs. Patients were classified *a priori* into the following diagnostic categories: sepsis, brain injury, respiratory failure, transplant, and postoperative cardiac surgery. Sepsis, septic shock, and systemic inflammatory response syndrome (SIRS) were defined using the critieria developed by the American College of Chest Physicians/ Society of Critical Care Medicine consensus [5]. All patients with sepsis and septic shock were classified as the Sepsis group. The mode of feeding, the nature of nutrition, and the caloric intake were also recorded. Children were classified as normal, chronically malnourished, or acutely malnourished. Chronic protein-energy malnutrition (CPEM) and acute protein-energy malnutrition (APEM) were defined by the Waterlow's stages [6]. Anthropometric measurements included midarm circumference (MAC), cutaneous triceps skin-fold thickness (TSF), midarm muscle circumference (MMC), midarm muscle area (MMA), and midarm fat area (MFA). Fat stores were assessed by measurements of TSF and MFA, and the somatic protein stores by MMC and MMA. Fat and protein stores were classified as normal, nutritionally at risk, or deficient as defined by Frisancho [7], Ryan and Martinez [8]. Normal values for weight for length and length for age were derived from the Standards of the National Center of Health Statistics [9]. Normal values for TSF, MAC, MMA, and MFA were derived from the Standards of the Ten-State Nutritional Survey [10]. The severity of illness was assessed by the Pediatric Risk of Mortality (PRISM) Score [11], the Therapeutic Intervention Scoring System (TISS) modified for children [12], and indices of organ failure. The multiple organ dysfunction score (MODS) grades increasing severity of dysfunction of six organ systems (cardiac, central nervous system, hematologic, hepatic, pulmonary, and renal) on a 4-point scale, so that a maximum of 24 points (100%) can be accrued. The highest scores for each organ at any point in the hospital course are added to derive the MODS [13], now recognized as a continuum of physiologic derangements, rather than an all-or-nothing phenomenon. The multiple organ system failure (MOSF) (at least two organ system failures, OSF) was defined using the criteria by Wilkinson et al. [14].

All patients were studied by indirect calorimetry within one week of acute stress if they remained mechanically ventilated. Oxygen consumption (VO_2_), carbon dioxide production (VCO_2_), and the respiratory quotient (RQ), which is the VCO_2_/VO_2_ ratio used for the substrate oxidation monitoring, were measured by indirect calorimetry using the Deltatrac Metabolic Monitor (SensorMedics) [15, 16]. The machine was calibrated before each measurement. Resting Energy Expenditure (REE) was calculated using the Weir formula modified by Frayn [17]. Predicted Basal Metabolic Rate (PBMR) was calculated using the Schofield equations [18]. Caloric, protein, fat, and carbohydrate intakes were calculated on the same day REE was determined. VCO_2_, VO_2_, REE, PBMR, and caloric intake were indexed to body surface area. We also calculated the ratio of REE/PBMR to determine the pattern of energy expenditure. An REE/PBMR ratios of >1.1, 0.9–1.1, and <0.9 were classified as hypermetabolic, normometabolic, and hypometabolic patterns, respectively. The ratio of caloric intake to REE was also calculated and ratios of >1.5 and <0.5 were classified as overfeeding and underfeeding, respectively.

### 2.3. Metabolic Markers

Blood samples were drawn on the same day of the metabolic study. The cytokines IL-10 and IL-6 were quantitated by commercially available ELISA technique. The other biochemical markers that were measured included acute phase reactants such as CRP and fibrinogen, as well as nutritional markers such as albumin, prealbumin, and transferrin. To aid interpretation of sequential measurements with changes in the magnitude of the acute-phase reaction, a quantitative way of monitoring the relationship between “nutritional” markers and acute proteins has been proposed—the Prognostic Inflammatory and Nutritional Index (PINI) [19], defined as PINI = CRP (mg/L) × a1-acid glycoprotein (mg/L)/Albumin (g/L) × Transthyretin (originally called prealbumin) (mg/L). However, because fibrinogen is more easily done in laboratories, and because albumin was found to be the worst indicator of nitrogen balance, we modified the PINI to the Nutritional Index (NI), as we had previously described [20]: NI = CRP (mg/dl) × Fibrinogen (mg/dl)/Transferrin (mg/dl) × Transthyretin (mg/dl). Outcome data were PICU Mortality status, Total hospital stay, PICU length of stay, and Duration of Mechanical Ventilation.

### 2.4. Statistical Analysis

Data were initially tested for normal distribution and homogeneity of variances by the Kolmogorov-Smirnov statistic with a Lilliefors significance level and the Levene test, respectively. Normally distributed data are expressed as mean ± SD, while nonnormally distributed data are given as median and range. Statistical analysis was performed with a two-tailed *t*-test for normally distributed unpaired data after Levene's correction for equality of variances or by Mann-Whitney U-Wilcoxon rank sum W test for nonnormally distributed data. Probability values of ≤.05 with two-tailed tests were considered significant. When a linear regression was calculated, Spearman's correlation coefficient was employed. Comparisons between nominal data were made by chi-square Pearson's statistic with continuity correction. To determine the strength of the association between the various metabolic patterns and mortality the relative risk estimation was measured. 

Differences among various disease groups and among subcohorts were tested by analysis of variance. Repeated measurements over time within and between groups were tested by analysis of variance for repeated measures. Multiple groups were tested by the one-way ANOVA with the Post Hoc Student-Newman-Keuls Multiple Range test. Whether the number of patients and measured variables differed between groups was tested by contingency table chi square analysis with continuity correction. 

Multivariate stepwise regression analysis was used to analyze the contribution of the PRISM, MOSF, MODS, SIRS, classified sepsis, probability of death values according to PRISM and MOSF [20] and various clinical factors, repleted substrate and substrate utilization, and malnutrition or anthropometry values to the variation in the inflammatory cytokines and other stress mediators. The Hosmer-Lemeshow Goodness-of-Fit Statistic was used in a logistic regression analysis model to compare the PRISM and MOSF probabilities of death to the mortality rate. Associations between NI or interleukin levels and anthropometry, severity of illness, clinical, or nutrition risk factor variables were examined by least squares linear regression for univariate analyses to identify exposure related risk factors for increased metabolism. Second, a stepwise linear multivariate regression was used to assess simultaneously the effect of each variable and to identify those factors that were independently associated with the magnitude of nutritional markers and stress mediators. Stepwise analysis was done with *P* < .1 as the criterion for eliminating variables (POUT) and *P* < .05 as the criterion for retaining variables (PIN). All analyses were done using the Statistical Package for the Social Sciences (SPSS) for Windows (release 17, SPSS, Chicago, IL) software package.

## 3. Results

### 3.1. Characteristics of the Patient Sample

A total of 77 patients were included in the study. The patients ranged in age from 1 to 210 months (median age 80 months). The mean length of MV was 6.7 ± 1.2 days (range 1 to 30 days), while the mean length of stay in PICU was 10 ± 1.6 days (range 1 to 41 days), and the mean hospital stay was 24.7 ± 4.3 days (range 3 to 133 days). Eighty-six percent of patients were Caucasian, with African American and Hispanics comprising 8% and 5% of the total study population, respectively. Male/female sex ratio was 57/20. There was no significant air-leak around the endotracheal tubes (mean difference between inhaled and exhaled VT was 0.03 ± 0.004%) and all patients were ventilated with a mean FiO_2_  0.4 ± 0.01 (range 0.25 to 0.6). The VO_2_/Body Surface Area (BSA) and VO_2_/BSA showed averages of 141.5 ± 28 (65–194 mL/min/m^2^) and 125.4 ± 27 (65.2–204 mL/min/m^2^), respectively. The RQ was 0.89 ± 0.01 (ranged from 0.73 to 1.07), the REE/BSA was 959.9 ± .01 (385–1435 kcal/m^2^/day), and the mean of REE/kg was 38.4 (16–63 kcal/kg/day). Fifty one patients (66%) had chronic diseases (cancer 17 patients, end organ failure 16, congenital heart disease or cardiomyopathy 12, metabolic or other genetic disease 6). Of all patients, 16 had undergone solid organ transplantation (20.8%), 12 were cardiac surgical patients (15.6%), 19 had sepsis (24.7%), 11 respiratory failure (14.3%), 10 metabolic coma (13.5%), 8 head injury (10.3%), and 1 patient suffered from severe Guillain-Barré syndrome (0.8%). The incidence of CPEM was 21.6% and the recorded risk for APEM was 8.1%. Regarding the regimens, patients were on at the time of inclusion in the study, 45.6% were on enteral nutrition, 36.4% on total parenteral nutrition (TPN), 14.3% on electrolyte and glucose intravenous solutions, and 3.9% on mixed enteranl parenteral diets. Nine patients died (11.7%), all with preexisted disease. The first day probability of death according to either PRISM (10.7%) or MOSF (9.7%) did not differ from the PICU mortality rate (Hosmer-Lemeshow Goodness-of-Fit Statistic).

### 3.2. Cytokines, Nutritional Indices, and Severity of Illness

Bivariate correlations between stress mediators or nutritional markers revealed the following.

1. PRISM was positively correlated to IL-10 (*r* *s* = 73, *P* < .000), CRP (*r* *s* = 40, *P* < .02), and IL-6 (*r* *s* = 37, *P* < .05).
2. TISS was positively correlated with IL-6 (*r* *s* = 0.54; *P* = .003), IL-10 (*r* *s* = 0.68; *P* < .001), CRP (*r* *s* = 0.52; *P* = .001), and NI (*r* *s* = 0.66; *P* < .001) but was negatively correlated with transthyretin (*r* *s* = −0.49, *P* < .003) and transferrin (*r* *s* = −0.72, *P* < .001).
3. The modified Nutritional Index correlated with IL-10 (*r* *s* = 0.48, *P* = .01), IL-6 (*r* *s* = 0.50, *P* = .01), and PRISM (*r* *s* = 0.51, *P* = .0001).
4. Significant negative correlations between IL-10 and transferrin (*r* *s* = −0.57, *P* < .002) and CRP and transthyretin (*r* *s* = −0.74, *P* < .000) were revealed.

In a multivariate analysis, PRISM was positively correlated with high IL-10 levels (*r* ^2^ = .34, *P* = .02) and negatively with transferrin (*r* ^2^ = −0.59, *P* = .0002). When a multiple linear regression analysis (stepwise method) was used, classified sepsis (*r* ^2^ = 0.25, *P* = .003), transferrin (*r* ^2^ = 0.21, *P* = .01), IL-6 (*r* ^2^ = 0.31, *P* = .002), and the NI (*r* ^2^ = 0.52, *P* = .001) were independently associated with the PRISM score (*F* = 12, *P* = .0000).

### 3.3. SIRS and Sepsis

**Figure 1:**
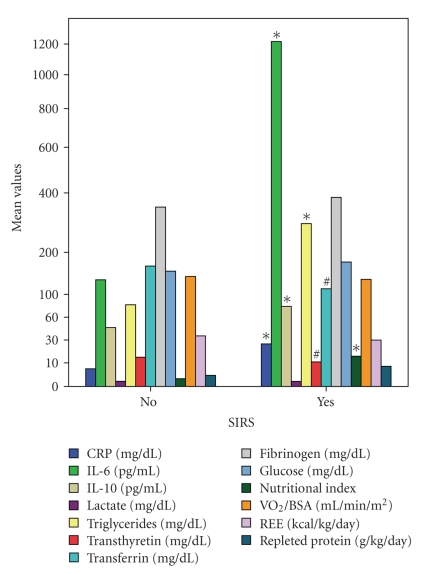
Comparison of metabolic monitor measurements, cytokines, and nutritional markers between patients with and without systemic inflammatory response syndrome (SIRS) (scale type power: exponent 0.5). In the presence of SIRS significantly increased (*) were: C-Reactive Protein (CRP), Interleukin-6 (IL-6), Interleukin-10 (IL-10), nutritional index, and triglycerides (*t*-test for unpaired data). Significantly decreased (#) despite a higher protein intake: transferrin and transthyretin. Did not differ compared to the non-SIRS patients: lactate, glucose, fibrinogen, Oxygen Consumption (VO_2_), and Resting Energy Expenditure (REE). BSA: Body Surface Area.

**Figure 2:**
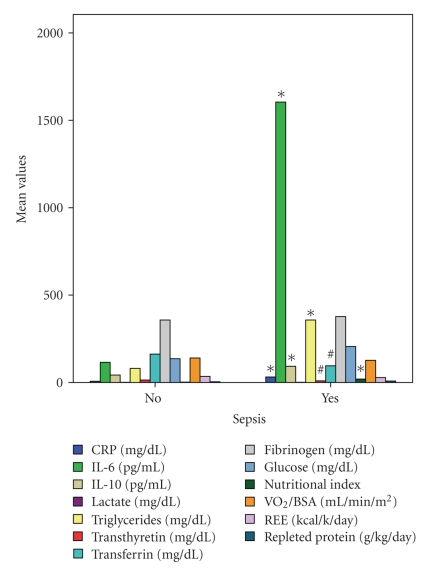
Comparison of metabolic monitor measurements, various cytokines, and nutritional markers between patients with and without sepsis (scale type power: exponent 0.5). Significantly increased (*) in the presence of sepsis: C-Reactive Protein (CRP), Interleukin-6 (IL-6), Interleukin-10 (IL-10), nutritional index, and triglycerides (*t*-test for unpaired data). Significantly decreased (#) despite a higher protein intake: transferrin and transthyretin. Did not differ compared to the nonsepsis patients: lactate, glucose, fibrinogen, Oxygen Consumption (VO_2_), and Resting Energy Expenditure (REE). BSA: Body Surface Area.

Compared to patients with no SIRS, patients with SIRS had elevated CRP (22 ± 15 mg/dl versus 5 ± 5 mg/dl, *P* < .0001), IL-6 (1277 ± 1675 versus 140 ± 77 pg/mL, *P* < .03), IL-10 (116 ± 129 versus 44 ± 18 pg/mL, *P* < .05), NI (11.2 ± 13 mg/dl versus 2.3 ± 2.4 mg/dl, *P* < .02), and triglycerides (352 ± 455 mg/dl versus 81 ± 26 mg/dl, *P* < .01) levels. But, transferrin (100 ± 41 versus 171 ± 33 mg/dl, *P* < .0001) and transthyretin (10 ± 4 versus 15 ± 6 mg/dl, *P* < .01) were lower in the presence of SIRS despite a higher protein intake (6 ± 4.5 versus 3.3 ± 3 g/kg/day, *P* < .005) (Figure 1). Similar results were observed in septic patients (Figure 2). Children with septic shock had had significantly higher levels of IL-6 (2659 ± 2206 versus 475 ± 981 pg/mL, *P* < .005) and IL-10 (219 ± 246 versus 63 ± 54 pg/mL, *P* < .007). Stepwise regression analysis (*F* = 12, *P* = .0002) showed that NI was positively correlated with SIRS (*r* ^2^ = .35, *P* = .007) and IL-6 (*r* ^2^ = 0.47, *P* = .01).

### 3.4. MODS-MOSF

**Figure 3:**
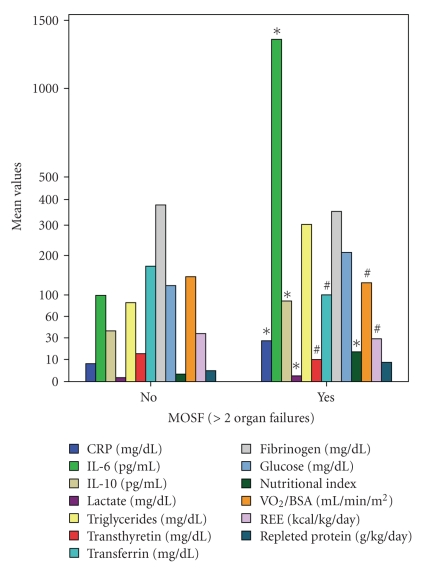
Comparison of metabolic monitor measurements, various cytokines, and nutritional markers between patients with and without multiple organ system failure (MOSF) (scale type power: exponent 0.5). Significantly increased (*) in the presence of MOSF: C-Reactive Protein (CRP), Interleukin-6 (IL-6), Interleukin-10 (IL-10), nutritional index, and lactate (*t*-test for unpaired data). Significantly decreased (#) despite a higher protein intake: Oxygen Consumption (VO_2_), Resting Energy Expenditure (REE), transferrin and transthyretin. Did not differ compared to the non-MOSF patients: triglycerides, glucose, fibrinogen. BSA: Body Surface Area.

The overall incidence of MOSF was 38.4% and the mean MODS 5 ± 3.4 (66.7% having had at least two organs dysfunctioning on a 24 scoring scale), respectively. Bivariate correlations showed that both IL-6 and IL-10 were positively correlated with the MODS (*r* *s* = 0.41, *P* < .03, and *r* *s* = 0.46, *P* < .02, resp.). In the presence of MOSF (>2 organ failures), CRP (23 ± 16 mg/dl versus 7.6 ± 8 mg/dl, *P* < .001), IL-6 (1394 ± 1686 versus 114 ± 74 pg/mL, *P* < .02), IL-10 (127 ± 129 versus 39 ± 16 pg/mL, *P* < .02), NI (14 ± 14 versus 2.1 ± 2.2, *P* < .003), and lactate (2.4 ± 2.2 mg/dl versus 1.1 ± .4 mg/dl, *P* < .02), but not glucose, triglycerides, or VCO_2_/BSA increased significantly compared to the non-MOSF patients, whereas VO_2_/BSA (133 ± 32 versus 148 ± 22 ml/min/m^2^, *P* < .02), REE (35 ± 12 versus 41 ± 10 kcal/kg/day, *P* < .03), transferrin (96 ± 36 versus 163 ± 41 mg/dl, *P* < .0001), and transthyretin (10 ± 4 versus 14 ± 6 mg/dl, *P* < .02) decreased significantly, despite a higher protein intake (6.4 ± 4.8 versus 3.6 ± 3 g/kg/day, *P* < .005) (Figure 3). Sepsis (odd ratio 5.24), among the clinical variables and IL-10 (odd ratio 1.24, 95% confidence interval (CI): 0.00–2.19) and transferrin (odd ratio 0.45, CI: 0.00–6.43) among the stress and nutritional mediators were independently associated with the development of MOSF (*P* < .0001). Metabolic and feeding patterns did not differ between patients with or without MOSF.

### 3.5. Metabolic Patterns

**Figure 4:**
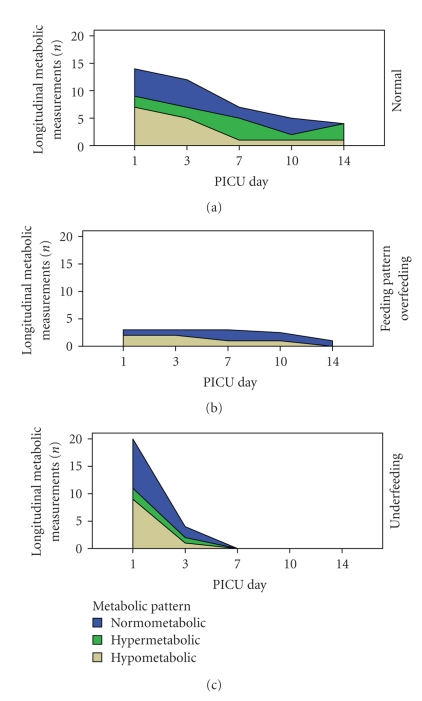
Longitudinal distribution of metabolic patterns in children during the acute phase of stress. The predominance of the hypometabolic pattern (50%) declined within 1 week of acute stress (20%) and the hypermetabolic patterns dominated only after 2 weeks (60%). Paired Resting Energy Expenditure (REE) and Predicted Basal Metabolic Rate (PBMR) measurements (*n* = 37). PICU: Pediatric Intensive Care Unit.

**Figure 5:**
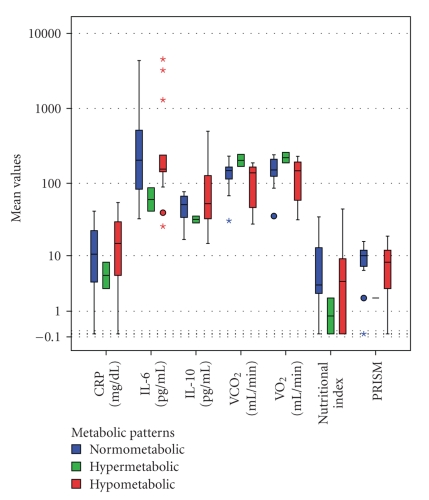
Only Oxygen consumption (VO_2_) and carbon dioxide production (VCO_2_) differed significantly among metabolic patterns (ANOVA, Bonferroni post-hoc tests, *P* < .0001). None of the cytokines and nutritional markers differed between the metabolic patterns. Note the (nonsignificant) lower trend of the Pediatric Risk of Mortality Score (PRISM) and C-Reactive Protein (CRP) values in the hypermetabolic group of patients. The Box-whisker plots show the median (horizontal line within the box) and the 10th and 90th percentiles (whiskers). The box length is the interquartile range (logarithmic scale). Solid circles represent outliers, stars extremes. IL-6: Interleukin-6, IL-10: Interleukin-10.

On the first day of stress, 48.6% of children were hypometabolic, 40.5% normometabolic, and only 10.8% hypermetabolic. In the series of longitudinal measurements of the 37 patients, the predominance of the hypometabolic pattern declined within 1 week of acute stress (20%) and the hypermetabolic pattern only emerged after 2 weeks (60%) (Figure 4). Although there was a trend for lower levels of cytokines and indices of severity of illness among hypermetabolic patients (Figure 5), only VO_2_ and VCO_2_ were independently associated with a hypometabolic pattern (*P* < .0001). There was increased incidence of hypometabolic pattern in patients with MOSF (51.7% versus 37.9%) or septic shock (57.1% versus 35.7%), which however did not reach statistical significance, and increased incidence of hypermetabolic pattern in patients with APEM (33% versus 16.4%, *P* < .004) or muscle stores depletion (66.7% versus 10%, *P* < .001). There was no relation between metabolic patterns and disease groups, SIRS, steroids, blocking agents, and gender.

### 3.6. Mortality

**Figure 6:**
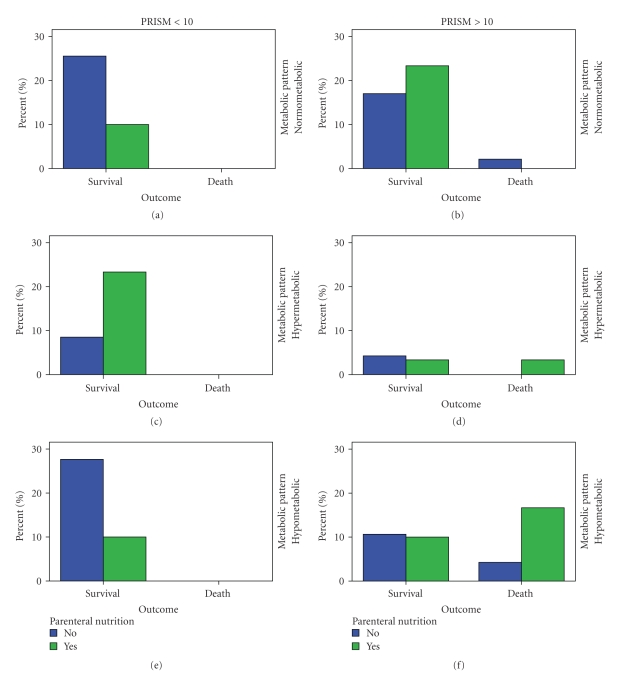
Mortality was (i) significantly higher (Pearson chi-square, asymptomatic significance 2-sided *P* < .05) in the hypometabolic pattern (22.6%) compared to the hypermetabolic (6.7%) or normometabolic patterns (3.2%), (ii) exclusively restricted to patients with Pediatric Risk of Mortality Score (PRISM)>10 (25.7% versus 0, *P* < .0001), and (iii) increased among those receiving partial or Total Parenteral Nutrition (TPN) (20% versus 6.4%, *P* = .7).

**Figure 7:**
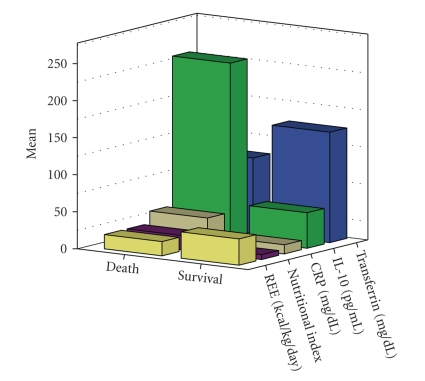
Opposite trends of C-Reactive Protein (CRP), Interleukin-10 (IL-10), and nutritional index compared to transferrin, and Resting Energy Expenditure (REE) were recorded between survivors and non-survivors.

Mortality was (a) higher (*P* < .05) in the hypometabolic pattern (22.6%) compared to the hypermetabolic (6.7%) or normometabolic (3.2%) patterns (Figure 6), (b) increased among those receiving partial or TPN (20% versus 6.4%, *P* = .07), (c) increased in patients with NI>10 (50% versus 7.2%, *P* = .005). Among nonsurvivors, CRP (*P* < .03), IL-10 (*P* < .0001), and NI (*P* < .003) increased significantly compared to survivors, whereas transferrin (*P* < .0001) and REE (*P* < .02) decreased significantly (Figure 7). In a logistic regression model of forward stepwise (conditional) analysis, only high IL-10 levels (odd ratio 1.38; CI: 0.00–1.75, *P* < .0001) and low measured REE (odd ratio 0.02; CI: <0.000, *P* < .0001) were independently associated with mortality.

### 3.7. Cytokines, Nutritional Indices, and Feeding Patterns

**Figure 8:**
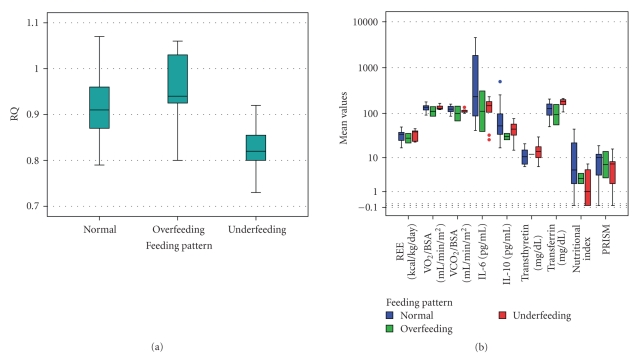
(a) Only the respiratory quotient (RQ) varied at higher levels in the overfeeding compared to the other groups (ANOVA, Bonferroni post-hoc tests, *P* < .0001); (b**)** Nutritional and stress indices did not differ among feeding groups during the acute phase of stress. The Box-whisker plots show the median (horizontal line within the box), and the 10th and 90th percentiles (whiskers). The box length is the interquartile range (logarithmic scale). Solid circles represent outliers. REE: Resting Energy Expenditure, VO_2_: Oxygen Consumption, VCO_2_: Carbon Dioxide production, BSA: Body Surface Area, IL-6: Interleukin-6, IL-10: Interleukin-10.

Malnourishment (APEM or CPEM), protein depletion (MMA score 2), and fat depletion (MFA score 2) did not affect NI values, IL-6 and IL-10 levels. Neither cytokines nor the NI differed among feeding groups (Figure 8), although RQ varied at higher levels in the overfeeding (0.91) compared to the other groups (.88 and  .89, resp., *P* < .0001). However, REE, VCO_2_/BSA, or VO_2_/BSA did not differ among feeding groups. REE was only correlated with the IL-10 (*r* ^2^ = −.48, *P* = .01) and white blood cell count (*r* ^2^ = .36, *P* = .001), but not PRISM. The NI was highly increased in the TPN group (11.8 ± 14) compared to either enteral nutrition alone (1.8 ± 2.19) or to various dextrose solution regimens (4.46 ± 5.34) (*F* = 2.86, *P* < .07). 

## 4. Discussion

### 4.1. Summary of Results

This is the first study that has examined the relationship between cytokines and metabolic patterns in pediatric patients with critical illness. In the current study, in response to stress, stress mediators increased and nutritional markers decreased proportional to the degree of illness severity. We further showed that only VO_2_ and VCO_2_ but not IL-6 or 10 were associated with a hypometabolic pattern which predominated the acute phase of stress. On the contrary, although in SIRS or sepsis the cytokine response was reliably reflected by increases in NI and triglycerides, it was different from the metabolic (VO_2_, VCO_2_) or glucose response. In MOSF, the metabolic work (VO_2_ and REE), transferrin, and transthyretin decrease irrespective of the feeding status. We also showed that a hypometabolic pattern represents an acute response to stress, is not influenced by activation of measured cytokines or feeding status, and is associated with increased mortality. Finally, our results indicate that the hypometabolic pattern predominates during the acute phase of stress turning out into a hypermetabolic pattern during convalescence.

### 4.2. Cytokines, Metabolism, and Severity of Illness, SIRS, or Sepsis

The NI used in this study readily reflects the metabolic response to stress in our patients, showing that priorities in liver synthesis are modified, with preferential production of newly synthesized hepatic glycoproteins classified as acute-phase reactants (such as CRP and fibrinogen) and inhibition of nutritional markers (such as transferrin and prealbumin albumin synthesis) [21]. Those changes are driven by a combination of counterregulatory hormones and the direct and indirect actions of the various inflammatory mediators, prostaglandin, and kallikreins during the acute phase of stress [22]. Recent studies have documented elevated levels of IL-1 beta, IL-6, IL-7, IL-8, IL-10, IL-13, and tumour necrosis factor-alpha in septic shock patients than in those with severe sepsis [23]. In terms of predicting mortality, the cytokines IL-1 beta, IL-4, IL-6, IL-8, and granulocyte colony-stimulating factor had good accuracy for predicting early mortality (<48 hours), whereas IL-8 and monocyte chemoattractant protein-1 had the best accuracy for predicting mortality at 28 days.Our results verify findings of a recent study in adult patients in which IL-6 and IL-10 were the key cytokines in the pathogenesis of severe sepsis; IL-6 was comparatively more associated with septic shock and IL-10 was comparatively more associated with mortality [24]. At the time sepsis was first suspected in neonates, IL-10 had a sensitivity of 17% and a diagnostic specificity of 99% [25]. What we further showed in this study was that not only interleukins, stress markers, the NI, and also triglycerides are increased in patients with sepsis and even SIRS, but that they are also positively correlated to the severity of illness. On the contrary, we could not verify that glucose, VO_2_, or VCO_2_ increase in the presence of SIRS and sepsis [26] in our non-diabetic population [27], but that only nutritional markers decrease significantly.

### 4.3. Cytokines, Metabolism, and MOSF

Previous studies showed that IL-6 and IL-8 concentrations during the first 24 hours were predictive of worsening organ dysfunction [3] or failure of organ dysfunction to improve on day three [23]. Because cytokines are influenced by a multitude of other factors occurring in the intensive care setting, [28, 29] regression analysis was done to find out factors independently associated with a specific cytokine induction or significantly influencing the development of MOSF. In our study, IL-6 and 10, the NI, and lactate increased with MOSF and nutritional indices decreased, but only sepsis and IL-10 among the stress mediators were independently associated with the development of MOSF. This antiinflammatory approach to MOSF along with the absence of an independent association with the proinflammatory IL-6 might be explained by an immediate hyperactivation of circulating monocytes, which might be rapidly followed by a substantial paralysis of cell function [30]. Although it has been previously shown that admission IL-10 is related to the severity of organ failure and mortality in children with septic shock [31], our results for the first time demonstrate that metabolism, as expressed by measured VO_2_ and REE, is down-regulated in patients with MOSF (Medline search).

### 4.4. Metabolic Patterns and MOSF

An important finding of this study is that the predominant metabolic pattern during the acute phase of critical illness is the hypometabolic (half of patients), whereas the hypermetabolic pattern only emerges during convalescence. Simultaneously, the most important factor that was leading to a hypometabolic response was MOSF or septic shock and, if persisted, impeding death (mortality). Previous studies have also demonstrated that children do not become hypermetabolic during critical illness [32] and that energy expenditure is close to or even lower than predicted basal metabolic rate [33]. The hypometabolic response may therefore reflect failure of the neurohumoral system to servoregulate the metabolic machinery, resulting in failure of energy production, reduced tissue substrate utilization and VO_2_ anaerobic metabolism and lactic acidosis, and ultimately organ failure. Growing evidence suggests that mitochondrial inhibition plays a major role in the development of multiple organ failure during sepsis [34]. Once mitochondrial dysfunction has developed, the regulated induction of a hypometabolic state, analogous to hibernation, may protect the cells from severe bioenergetic failure and a critical fall in ATP. Though this is clinically manifest as organ dysfunction, it may actually represent an adaptive response to a prolonged, severe inflammatory stress [34]. These changes suggest a progressive failure of energy production, probably at the level of the mitochondria, resulting in reduced tissue oxygenation, decreased substrate utilization, and ultimately MOSF and death. This hypothesis is further supported by findings of this study, which have shown a significant relationship of MOSF to the NI followed by depressed VO_2_ and nutritional markers.

### 4.5. Metabolic Patterns and Feeding Status

Our MOSF or SIRS patients were receiving higher amounts of protein compared to the non-MOSF or non-SIRS patients, so that we were unable to assess the contribution of relative protein depletion to the organ nutrition derangement. Thus, food restriction, where all essential nutrients were reduced in proportion, was a physiologic stress that, while limiting growth, did not activate or impair the systemic inflammatory response, whereas a very low protein diet with little change in energy intake had a substantial impact on systemic inflammation, body composition, and growth [35]. 

It has been previously shown that caloric restriction inhibits up-regulation of inflammatory cytokines and TNF-alpha, and activates IL-10 and haptoglobin in the plasma of streptozotocin-induced diabetic rats [36]. Although it has been also shown that the expression levels of IL-10 were higher and IL-6 genes were expressed less in malnourished noncritically ill children [37], we did not find that malnourishment or severe substrate depletion affected key cytokines reaction or metabolism. We had also previously shown that patients at risk for protein stores depletion had a higher incidence of MOSF, whereas patients with fat stores depletion had a higher probability of death, when compared with nutritionally normal children [33]. 

In this study, we showed an increased incidence of hypermetabolic pattern in patients with APEM or muscle stores depletion, an indicative of the severe hyper-catabolic state in which these groups of patients are. In addition, although metabolic patterns were not related to specific feeding patterns, overfed patients had significantly higher RQ, supporting RQ usefulness to identifying overfeeding and excluding underfeeding [38].

### 4.6. Cytokines, Metabolism and Nutritional Interventions

Regarding fats, high-density lipoproteins have been shown to bind and neutralize lipopolysaccharide and are regarded as possible therapeutic agents for sepsis and conditions associated with local or systemic inflammation [39]. However, in recent years, a multitude of possible immunomodulatory properties other than lipopolysaccharide neutralization have become evident [40]. Thus, under stress conditions, glutamine availability can become rate limiting for key cell functions, such as phagocytosis and antibody production [41]. In addition, the reprioritization of the liver synthetic pathways and the increased catabolism of certain of these proteins make them much more an indicator of severe illness than a measure of the liver's synthetic ability [42, 43]. In ill patients, like those in our study, plasma concentrations of visceral proteins tend to be lowered and return to normal as the disease process itself recovers [44]. By day 5, nutritional indices and antioxidant catalysts showed a higher increasing trend in an immune-enhancing formula compared with conventional early enteral nutrition [45]. However, we did not use any immune-enhancing formula in the present study.

The highest mortality in the partial or TPN group might be related to the worst severity of disease, as evidenced by a PRISM>10, and to the worst inflammation, as evidenced by an NI>10 among these patients. However, our data also suggested that parenteral nutrition might be associated with a worse effect on metabolism than enteral nutrition. Recent work has shown that up-regulation of Toll-like receptors in intestine in response to TPN administration and a lack of enteral nutrition may be associated with an increased risk of septic shock due to bacterial translocation caused by a significant decline in intestinal intraepithelial lymphocyte-derived IL-10 expression, a cytokine that has been shown in vitro to maintain tight junction integrity [46], and by interferon gamma-mediated intestinal epithelial cell apoptosis [47]. Comparing the effects of early randomized immune or nonimmune-enhancing enteral nutrition on cytokine production in children with septic shock, we had previously showed that IL-6 levels were significantly lower in the immune-enhancing than in the non-immune-enhancing group [48]. Similarly, in a multivariate regression analysis among children with severe head injury, we had showed that IL-8 was independently negatively correlated with immunonutrition [43].

### 4.7. Limitations

The main limitation of the study is that it only examined the acute phase of the critical illness in an intensive care setting. More extended studies are needed in children and adults or various subcohorts [49] to assess metabolic patterns related to cytokine reactions at different time points in their illnesses. An indication of such a need might be the longitudinal series we performed that produced a different metabolic pattern during the acute stress phase compared to a presumed (2nd week) convalescent phase of the illness. Although we did not attempt to control variables such as diagnosis, severity of illness, type of patients, or the care provided during the study, we entered all these variables in multiple regression and logistic analyses of our data. It is difficult to determine with accuracy, however, whether these factors affected our results.

## 5. Conclusions

In summary, more than half of the patients had energy expenditure more than 10% above or below predicted values and an associated increased risk of mortality during an acute illness. In the current study, in response to stress, interleukins and stress mediators increased and nutritional markers decreased proportional to the degree of illness severity and development of SIRS, sepsis, or MOSF. However, only VO_2_ and VCO_2_ but not IL-6 or 10 were associated with a hypometabolic pattern which predominated the acute phase of stress. On the contrary, although in SIRS or sepsis the cytokine response was reliably reflected by increases in NI and triglycerides, it was different from the metabolic (VO_2_, VCO_2_) or glucose response. A metabolic work (VO_2_ and REE) derangement, like the hypometabolic pattern recorded in MOSF, developed irrespective of the feeding status, was not influenced by activation of measured cytokines, and had a worse outcome. Since different metabolic patterns might be interchanging during a clinical course of severe illness, a systemic followup of metabolism might improve early diagnosis and contribute to an optimal therapeutic support. Further research to address possible pharmaconutritional interventions in the various phases of the metabolic response to stress should be carefully planned.
